# 3D *in vivo* Magnetic Particle Imaging of Human Stem Cell-Derived Islet Organoid Transplantation Using a Machine Learning Algorithm

**DOI:** 10.3389/fcell.2021.704483

**Published:** 2021-08-12

**Authors:** Aixia Sun, Hasaan Hayat, Sihai Liu, Eliah Tull, Jack Owen Bishop, Bennett Francis Dwan, Mithil Gudi, Nazanin Talebloo, James Raynard Dizon, Wen Li, Jeffery Gaudet, Adam Alessio, Aitor Aguirre, Ping Wang

**Affiliations:** ^1^Precision Health Program, Michigan State University, East Lansing, MI, United States; ^2^Department of Radiology, College of Human Medicine, Michigan State University, East Lansing, MI, United States; ^3^Lyman Briggs College, Michigan State University, East Lansing, MI, United States; ^4^Department of Orthopedics, Beijing Charity Hospital, Capital Medical University, Beijing, China; ^5^Medgar Evers College, City University of New York, Brooklyn, NY, United States; ^6^Department of Neuroscience, College of Natural Science, Michigan State University, East Lansing, MI, United States; ^7^College of Natural Science, Michigan State University, East Lansing, MI, United States; ^8^Department of Chemistry, College of Natural Science, Michigan State University, East Lansing, MI, United States; ^9^Department of Radiology, UT Southwestern Medical Center, Dallas, TX, United States; ^10^Department of Electrical and Computer Engineering, College of Engineering, Michigan State University, East Lansing, MI, United States; ^11^Institute for Quantitative Health Science and Engineering (IQ), Department of Biomedical Engineering, Michigan State University, East Lansing, MI, United States; ^12^Magnetic Insight Inc., Alameda, CA, United States; ^13^Department of Computational Mathematics, Science and Engineering, College of Engineering, Michigan State University, East Lansing, MI, United States

**Keywords:** artificial intelligence, unsupervised machine learning, magnetic particle imaging, stem cell tracking, diabetes

## Abstract

Stem cell-derived islet organoids constitute a promising treatment of type 1 diabetes. A major hurdle in the field is the lack of appropriate *in vivo* method to determine graft outcome. Here, we investigate the feasibility of *in vivo* tracking of transplanted stem cell-derived islet organoids using magnetic particle imaging (MPI) in a mouse model. Human induced pluripotent stem cells-L1 were differentiated to islet organoids and labeled with superparamagnetic iron oxide nanoparticles. The phantoms comprising of different numbers of labeled islet organoids were imaged using an MPI system. Labeled islet organoids were transplanted into NOD/scid mice under the left kidney capsule and were then scanned using 3D MPI at 1, 7, and 28 days post transplantation. Quantitative assessment of the islet organoids was performed using the *K-means*++ algorithm analysis of 3D MPI. The left kidney was collected and processed for immunofluorescence staining of C-peptide and dextran. Islet organoids expressed islet cell markers including insulin and glucagon. Image analysis of labeled islet organoids phantoms revealed a direct linear correlation between the iron content and the number of islet organoids. The *K-means*++ algorithm showed that during the course of the study the signal from labeled islet organoids under the left kidney capsule decreased. Immunofluorescence staining of the kidney sections showed the presence of islet organoid grafts as confirmed by double staining for dextran and C-peptide. This study demonstrates that MPI with machine learning algorithm analysis can monitor islet organoids grafts labeled with super-paramagnetic iron oxide nanoparticles and provide quantitative information of their presence *in vivo*.

## Introduction

Type 1 diabetes (T1D) is characterized by an absolute deficiency of insulin secretion with hyperglycemia as a consequence. Transplantation of exogenous pancreatic islets to replace dead or dysfunctional endogenous beta cells is a strategy for controlling blood glucose levels in T1D patients. Severe shortage of cadaveric organ donors, requirement for lifelong immunosuppression, and reverting to using insulin after transplantation, however, largely hampers its application. In recent years advances in stem cell research, such as the derivation of human induced pluripotent stem cells (hiPSCs) and the emergence of organoid technologies, have enabled the creation of highly sophisticated human tissues and organ-like structures *in vitro*. These tissues could be used for isogenic or allogenic transplantation in the clinical setting to treat a diverse number of conditions, including T1D. *In vitro* created pancreatic islet organoids can be readily generated from hiPSCs matching the patient, thus providing an alternative similar to cadaveric donor islets that bypasses immune rejection concerns. Additionally, the number of pancreatic islet organoids that can be potentially produced is unlimited. A significant hurdle, however, is monitoring integration and survival of the pancreatic islet organoid after transplantation, and the lack of suitable non-invasive imaging techniques allowing us to determine the longer-term graft outcome (Rizzo et al., 2017; Wang and Aguirre, 2018).

Stem cell replacement therapy needs a technique that can quantitatively evaluate the fate of cells *in vivo* with high specificity and sensitivity. There are several techniques that have been used for imaging of islet or stem cell transplantation, including: optical imaging, magnetic resonance imaging (MRI) and positron emission tomography (PET). Optical imaging methods, both bioluminescence and fluorescence imaging, have the limitation of penetration depth preventing linear quantification and clinical translation. PET has excellent tracer sensitivity and depth penetration but cannot assess cell fate due to the limited tracer half-life. The evaluation of islet transplantation efficacy in the clinic relies on destructive analytical methods like histology or functional improvements that can take months to manifest. Most studies of cell tracking have used super-paramagnetic iron oxide nanoparticles (SPIONs) labeled cells, because SPIONs-based methods have few effects on cell viability, proliferation, and differentiation (Crabbe et al., 2010; Wang and Moore, 2012; Janowski et al., 2014; Bulte and Daldrup-Link, 2018), along with excellent depth penetration and *in vivo* persistence on the order of months. The primary challenge for MRI-based SPIONs cell tracking, however, is that SPIONs induced MRI signal dropouts that are difficult to distinguish from tissues with naturally low MRI signal (e.g., bones, tendon, lungs, bleeding, or any tissues near air). Moreover, MRI methods with positive contrast suffer from toxicity and sensitivity challenges (Magnitsky et al., 2017).

**FIGURE 1:**
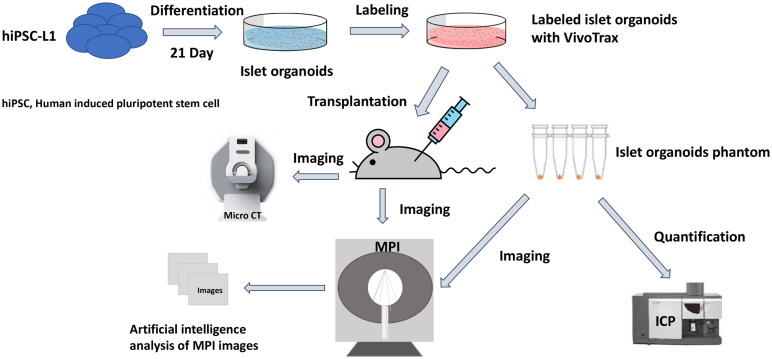
Scientific method flow chart of the study. The goal of this study is to test a novel imaging method magnetic particle imaging (MPI) for quantifying stem cell derived islet organoids phantoms *in vitro* and monitoring of transplanted islet organoids *in vivo*. An artificial intelligence algorithm had been applied for segmentation and quantification of nanoparticle labeled islet organoids in mouse model. This paves the way for MPI to be used in stem cells transplantation therapies for diabetes.

Magnetic particle imaging (MPI) is a novel imaging modality that directly detects the SPIONs, and is specific, sensitive, and linearly quantitative (Talebloo et al., 2020). MPI has been used for a wide range of biomedical applications such as tumors (Yu et al., 2017; Wu et al., 2019), vascular imaging (Khandhar et al., 2017), drug delivery (Wang et al., 2019), and *in vivo* tracking of labeled cells (Bulte et al., 2011; Zheng et al., 2015, 2016). Previously, our group demonstrated MPI could be used for *in vivo* tracking of transplanted pancreatic islets in a mouse model (Wang et al., 2018). Furthermore, we have developed an unsupervised machine learning (ML) algorithm for monitoring *in vivo* 2D MPI data of islet grafts (Hayat et al., 2020). Such algorithms are required for standardized, high-throughput monitoring of transplanted organoids *in vivo* due to the highly variable nature of a region of interest (ROI) between MPI data. Often, it is difficult for human raters to maintain consistency regarding the threshold of cutoff for the true MPI signal from background. Therefore, applying a ML algorithm for segmenting of the desired ROIs from an MPI scan provides a reliable and standardized method of image quantification. So far, there are no reports on using MPI for imaging stem cell-derived islet organoids and it is critical to advance our algorithm to a 3D setting for more accurate and reliable signal quantification. Herein, we demonstrate the feasibility to develop and use the *K-means*++ clustering-based, unsupervised ML algorithm to provide a novel method/tool for 3D image segmentation and quantification in the MPI domain, using SPIONs labeled stem cell differentiated islet organoids in a mouse model. We then used the segmentation output from the *K-means*++ algorithm to generate a standard curve using fiducial markers in order to estimate the total iron value (TIV) of segmented ROIs. This provides information on the SPIONs accumulation within the labeled islet organoids and permits longitudinal evaluation of TIV of the ROI in the mice (Figure 1).

## Materials and Methods

### Stem Cell Culture and Pancreatic Islet Organoid Differentiation

All hiPSC lines were validated for pluripotency and genomic integrity. hiPSC-L1 were cultured in Essential 8 Flex medium (Thermo Fisher Scientific, MA, United States) containing 1% penicillin/streptomycin (Gibco, Thermo Fisher Scientific, MA, United States) on six-well plates coated with growth factor-reduced Matrigel (Corning, NY, United States) in an incubator at 37°C, 5% CO_2_, until 60–80% confluency was reached, at which point cells were split into new wells using ReLeSR passaging reagent (STEMCELL Technologies, Vancouver, BC, Canada).

To generate embryonic bodies, the confluent cultures were dissociated into small cluster suspension by incubation with accutase (Innovative Cell Technologies, Inc., San Diego, CA, United States). Cells were counted and each well of six-well low-adherence plates (Corning, NY, United States) were seeded with 5.5 × 10^6^ cells in 5.5 ml Essential 8 Flex Medium supplemented with 10 ng/ml Activin A protein (Activin A, R&D Systems, MN, United States) and 10 ng/ml heregulin-beta-1 (PeproTech, NJ, United States). Plates were placed on an orbital shaker at 100 rpm to induce embryonic bodies.

To generate islet organoids, cell differentiation underwent five stages (21 days) was performed as described before (Russ et al., 2015). Briefly, to initiate differentiation, embryonic body was suspended into a low-adherence six-well plate in 5.5 ml d1 media [RPMI (Gibco) containing 0.2% FBS, 1:5,000 insulin-transferrin-selenium (ITS) supplement (Gibco), 100 ng/ml activin A, and 50 ng/ml WNT3a (R&D Systems)]. Media thereafter were changed daily, by removing either 4.5 ml media (at the end of d1) or 5.5 ml media the following days and adding back 5.5 ml fresh media until day 9. After day 9, only 5 ml of media was removed and added daily (Russ et al., 2015). Media in this differentiation protocol consist of the following: d2: RPMI containing 0.2% FBS, 1:2,000 ITS, and 100 ng/ml activin A; d3: RPMI containing 0.2% FBS, 1:1,000 ITS, 2.5 μM TGF-beta Inhibitor IV (Calbiochem, MA, United States), and 25 ng/ml KGF (R&D Systems); d4–5: RPMI containing 0.4% FBS, 1:1,000 ITS, and 25 ng/ml KGF; d6–7: DMEM (Gibco) with 25 mM glucose containing 1:100 B27 (Gibco), 3 nM TTNPB (Sigma); d8: DMEM with 25 mM glucose containing 1:100 B27, 3 nM TTNBP, and 50 ng/ml EGF (R&D Systems); d9: DMEM with 25 mM glucose containing 1:100 B27, 50 ng/ml EGF, and 50 ng/ml KGF; d10–14: DMEM with 25 mM glucose containing 1:100 B27, 500 nM LDN−193189 (Stemgent, MD), 30 nM TPB (Millipore Sigma, MA, United States), 1,000 nM ALKi II (Axxora), and 25 ng/ml KGF; and d15–21: DMEM with 2.8 mM glucose containing 1:100 Glutamax (Gibco) and 1:100 non-essential amino acids solution (Gibco) (Russ et al., 2015).

### Immunofluorescence Confocal Microscopy Imaging

Human islet organoids were transferred to microcentrifuge tubes (Eppendorf) using a cut 1000-mL pipette tip to avoid disruption to the islet organoids and fixed in 4% paraformaldehyde solution for 15 min at room temperature. Fixation was followed by three washes in PBS and incubation in blocking solution (10% goat normal serum in PBS) on a thermal mixer (Thermo Scientific) at 300 RPM at 4°C overnight. Islet organoids were then incubated with anti-insulin primary antibody (Abcam, Cambridge, MA, United States) and anti-glucagon antibody (Abcam, Cambridge, MA, United States) in Antibody Solution (10% goat normal serum and 0.5% bovine serum albumin in PBS) on a thermal mixer at 300 RPM at 4°C for 24 h. Primary antibody exposure was followed by three washed in PBS and incubation with FITC-labeled goat anti-mouse secondary IgG (Abcam, Cambridge, MA, United States) and Texas red conjugated goat anti-rabbit secondary IgG (Santa Cruz Biotechnology, Santa Cruz, CA, United States) in Antibody Solution on a thermal mixer at 300 RPM at room temperature for 1 h in the dark. The stained islet organoids were washed three times in PBS before being mounted on glass microscope slides (Fisher Scientific) using mounting medium containing DAPI (Vectashield; Vector Laboratories). Ninety micrometers Polybead Microspheres (Polysciences, Inc.) were placed between the slide and the coverslip (No. 1.5) to preserve some of the 3D structure of the organoids while accommodating the penetration capacity of the confocal microscope. Samples were imaged using an Olympus FluoView 1000 Filter-based laser scanning confocal microscope. Immunofluorescence images of islet organoids were semi-quantitatively analyzed using Fiji.^1^ Briefly multi-color fluorescent images were split into single channels and converted to grayscale images. The area of interests was selected using selection tools in Fiji. The insulin and glucagon positive cells percentages of islet organoids were calculated.

### Glucose-Stimulated Insulin Secretion Assay

One Hundred islet organoids (between 28 and 30 days of differentiation) were pre-incubated in 1 ml of low (2.8 mM) glucose Krebs buffer (KRB) and washed twice with KRB to remove residual insulin, then islet organoids were incubated in 1 ml of 2.8 mM glucose KRB for 30 min in a 37°C cell culture incubator, and the supernatant was collected. After they were washed twice with 2.8 mM glucose KRB, the islet organoids were incubated in 1 ml of high (28 mM) glucose Krebs buffer for 30 min, and the supernatant was collected. Human insulin was measured using the Insulin Human ELISA kit (Abcam, ab100578). Human insulin measurements were normalized by cell counts that were acquired by dispersing islet organoids into single cells with Accutase (Innovative Cell Technologies, Inc.) and counted using a cell counter.

### Cell Labeling and *in vitro* Characterization of Labeled Cells

Islet organoids were feed with the concentration of 560 μg/ml VivoTrax (Magnetic Insight Inc., Alameda, CA, United States) in CMRL media with 5% FBS and incubated for 48 h at 37°C with 5% CO_2_ (Wang et al., 2018; Hayat et al., 2020). VivoTrax is a dextran coated SPION with core size of 4.2 nm and a mean hydrodynamic diameter of 62 nm. Cell labeling efficiency was tested using immunofluorescence staining for dextran coating with anti-dextran antibody (STEMCELL Technologies, Vancouver, BC, Canada), followed by an FITC-labeled goat anti-mouse secondary IgG (Abcam, Cambridge, MA, United States), mounted with a mounting medium containing DAPI and analyzed using fluorescence microscopy. Islet organoids viability was tested after labeling by colorimetric [3-(4,5-dimethylthiazol-2-yl)-2,5-diphenyltetrazolium bromide] assay according to the manufacturer’s protocol (Promega, Madison, WI, United States) (Wang et al., 2011, 2012).

Cell function of secreted C-peptide was also tested using immunofluorescence staining with anti-C-peptide primary antibody (Abcam, Cambridge, MA, United States) and anti-dextran antibody (STEMCELL Technologies, Vancouver, BC, Canada), followed by an FITC-labeled goat anti-mouse secondary IgG (Abcam, Cambridge, MA, United States) and Texas red conjugated goat anti-rabbit secondary IgG (Santa Cruz Biotechnology, Santa Cruz, CA, United States).

### Imaging of Islet Organoid Phantoms

*In vitro* phantoms comprising of different numbers of VivoTrax labeled islet organoids (0, 25, 50, 100, 200, and 400) in PBS were imaged using an MPI scanner (MOMENTUM MPI, Magnetic Insight Inc., Alameda, CA, United States) with the fiducial markers. Each 2D MPI images were acquired with parameters of a field-of-view (FOV) of 6 cm × 12 cm, a 5.7 T/m selection field gradient, a drive field strength of 20 mT peak amplitude and a 45.0 kHz drive frequency. Images were reconstructed using x-space reconstruction. Quantification of the islet organoids phantoms was performed using the 2D MPI image intensity calibrated against a fiducial marker of known iron content (2.2 μg of iron) using VivoQuant imaging software (Invicro, Boston, MA, United States).

### Inductively Coupled Plasma Optical Emission Spectroscopy

Iron content of labeled islet organoids were determined using inductively coupled plasma optical emission spectroscopy (ICP-OES). Different numbers of labeled islet organoids were digested with concentrated nitric acid (Sigma-Aldrich, St. Louis, MO, United States). Digested samples were diluted and the total iron content of the samples were determined using an Agilent 710-ES ICP-OES (Agilent Technologies, Santa Clara, CA, United States). For the measurement of the iron content with ICP-OES, a calibration line was generated by six standard samples containing 0, 0.125, 0.25, 0.5, 1, 2, 3, and 4 ppm Fe. All iron standards were prepared from a stock certified standard reference material (FeCl_3_, Iron Standard for ICP, 1000 ± 2 mg/l Fe in 2% nitric acid, Sigma-Aldrich, St. Louis, MO, United States). An eight-point calibration curve was performed prior to sample analysis. The total iron content of samples was calculated, accounting for the number of islet organoids provided as well as dilution factors, as the mean value for analysis.

### Cell Transplantation Under Kidney Capsule in a Mouse Model

All animal experiments were performed in compliance with institutional guidelines and were approved by the Institutional Animal Care and Use Committee (IACUC) at the Michigan State University. Labeled islet organoids were collected 1 h before transplantation. Female NOD/scid immunodeficient mice (*n* = 5, 10 weeks old, Jackson Laboratory, Bar Harbor, ME, United States) were anesthetized with 2% isoflurane. An incision made to expose the left kidney of the mouse, a catheter needle inserted underneath the kidney capsule and 800 islet organoids VivoTrax labeled were transplanted. Then the incision was closed, mice were monitored after transplantation.

### MPI Tracking of Transplanted Islet Organoids Under Kidney Capsule of Mice

Under anesthesia with 2% isoflurane, mice were imaged using 3D MPI at 1, 7, and 28-day post-transplantation (*n* = 3) with operating parameters, a FOV of 6 cm × 6 cm × 12 cm, acquisition time of 10 s per projection (total 55 projections) plus 30 s for automatic set up of the magnets, with an approximate total time of 35 min including for image reconstruction. Anatomic CT reference images were acquired by the whole-body model of Perkin Elmer QuantumGX microCT. MPI images were co-registered to CT with fiducial markers using VivoQuant Imaging Software (Invicro, Boston, MA, United States). Control animals did not receive islet organoids (*n* = 2).

### 3D *K-means*++ Unsupervised Machine Learning Algorithm

In order to segment the ROI from the MPI, we used the previously established *k-means*++ algorithm for ROI segmentation and a standard curve model for prediction of the TIV of the cells (Hayat et al., 2020). This TIV correlates linearly to increasing ROI intensity and size (Hayat et al., 2020). Hence, here we apply the *k-means*++ algorithm to a 3D MPI Image sequence through layer-by-layer segmentation of the individual ROIs and TIV estimations from 32 to 36 layers per scan, and these values are summed to provide a TIV for the entire 3D structure. As determined in our previous study by the elbow method, the *k*-value for number of centroids in this study was 4 (Hayat et al., 2020). The concentrations of the fiducial markers (1 μl) used to generate the standard curve were 10, 20, and 40% of VivoTrax solution (5.5 mg/ml iron). For all MPI image scans, placement of fiducial markers was kept in the same location in order to provide optimal data for *k-means*++ segmentation of the ROI and subsequent TIV estimation *via* the generated standard curve.

### Intraclass Correlation Coefficient Validation

In order to measure accuracy of the algorithm and determine its validity, its TIV prediction was compared to that of a manual rater, in this case a board-certified radiologist. The rater segmented the ROI manually on 15 images using the VivoQuant Imaging Software (Invicro, Boston, MA, United States), and total pixel sum values were extracted for TIV analysis from the ROIs using a ratio method with reference to a singular fiducial marker for TIV prediction (Makela et al., 2020), calibrated against a 40% fiducial marker of known iron concentration (2.2 μg of iron). For statistical analysis, SPSS statistical software (IBM, Armonk, NY, United States) was used to calculate intraclass correlation coefficient (ICC), which provides a measure of the inter-rater reliability between the rater and algorithm. A two-way mixed model with a confidence interval of 95% was selected, and a measure of absolute agreement was calculated with the ICC. A higher ICC score indicates greater reliability of algorithm performance due to the high degree of agreement between the rater and algorithm.

### *Ex vivo* Immunohistochemistry

After the last round of MPI, animals were sacrificed and left kidneys were removed, fixed in 4% paraformaldehyde solution for 16 h at 4°C, and embedded in paraffin. Paraffin sections of grafts under the left kidney capsule were incubated with anti-C-peptide primary antibody (Abcam, Cambridge, MA, United States) and anti-dextran antibody (STEMCELL Technologies, Vancouver, BC, Canada) at 4°C for 16 h, followed by an FITC-labeled goat anti-mouse secondary IgG (Abcam, Cambridge, MA, United States) and Texas red conjugated goat anti-rabbit secondary IgG (1:100 dilution, Santa Cruz Biotechnology, Santa Cruz, CA, United States) at room temperature for 1 h. All sections were mounted with a mounting medium containing DAPI (Vectashield; Vector Laboratories) and analyzed using fluorescence microscopy (Eclipse 50i; Nikon Metrology, Brighton, MI, United States) (Pomposelli et al., 2020; Wang et al., 2020).

### Statistical Analysis

Data are presented as mean ± SD. Statistical comparisons between two groups were evaluated by Student *t*-test and corrected by one-way ANOVA for multiple comparisons using GraphPad Prism 5 (GraphPad Software, Inc., La Jolla, CA, United States). A two-way repeated measures ANOVA was performed for evaluating TIV predictions as the independent variable for different imaging days. Correlation and linear regression analysis between measured iron content in the phantoms and the number of labeled islets was assessed using GraphPad Prism 5 and SPSS statistical software (IBM, Armonk, NY, United States) as well. A value of *p* < 0.05 was considered to be statistically significant.

## Results

### Characterization of Differentiated Islet Organoids

**FIGURE 2:**
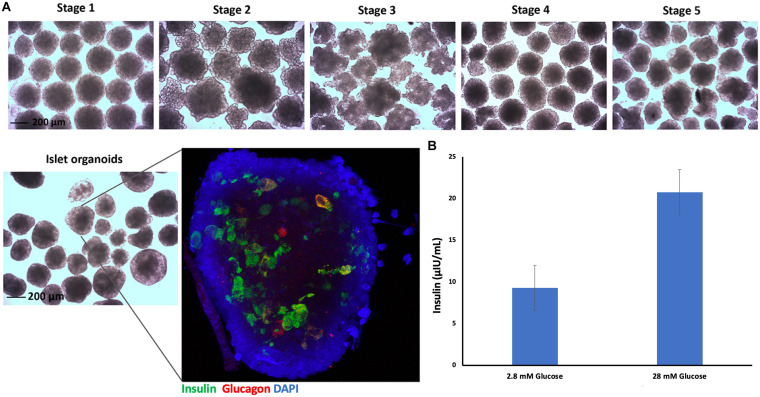
Islet organoids differentiation and characterization. **(A)** Micrographs of differentiated cell clusters at five stages under light microscopy, magnification bar = 200 μm. Immunofluorescence staining of differentiated islet organoids. Insulin (green), glucagon (red), and cell nuclei (DAPI, blue), magnification bar = 200 μm. **(B)** ELISA measurement of secreted human insulin from islet organoids stimulated with low and high glucose, with a 30-min incubation for each concentration (*n* = 3).

Embryoid bodies derived from hiPSC-L1 cells were produced in 3D-suspension culture to generate pancreatic islet organoids using already described protocols. After 21 days differentiated islet organoids were stained for immunofluorescence and confocal imaging to determine expression of insulin (24 ± 4%) and glucagon (9 ± 3%) in the differentiated islet organoids (Figure 2A and Supplementary Video 1). Functional test glucose-stimulated insulin secretion (GSIS) assay demonstrated the differentiated islet organoids reacted to glucose stimulation and secrete human insulin (Figure 2B). We further analyzed the expression of hormone genes in islet organoids including insulin and glucagon. Quantitative RT-PCR analysis showed high levels of insulin and glucagon gene transcripts in stage 5 cell clusters and islet organoids (Supplementary Figure 1), which was consistent with the immunofluorescence analysis.

### Assessment of Labeling Efficacy and Viability of VivoTrax Labeled Islet Organoids

**FIGURE 3:**
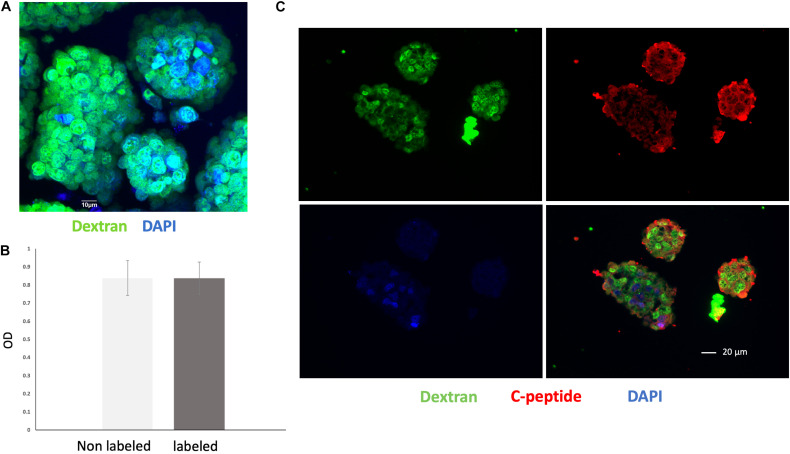
Cell labeling efficiency and viability assay. **(A)** Anti-dextran fluorescence immunostaining of islet organoids confirming successful VivoTrax labeling dextran coating (green), and cell nuclei (DAPI, blue), magnification bar = 10 μm. **(B)** MTT assay showed that VivoTrax labeling did not affect islet organoids viability compared with non-labeled control (*p* > 0.05). Data are represented as mean ± SD. **(C)** Double fluorescence immunostaining for dextran and C-peptide of VivoTrax labeled islet organoids dextran coating (green), C-peptide (red), and cell nuclei (DAPI, blue), magnification bar = 20 μm.

Nearly all of the cells within the islet organoids were labeled with VivoTrax as confirmed by anti-dextran antibody staining (Figure 3A). MTT assay showed that compared with non-labeled islet organoids, VivoTrax labeling treatment did not have significant effect on islet organoid viability (Figure 3B; *p* > 0.05). Double stained islet organoids with C-peptide (in green) and dextran showed that the VivoTrax labeled islet organoids expressed C-peptide, which demonstrated the labeling treatment did not affect insulin biosynthesis in differentiated islet organoids (Figure 3C).

### Imaging of Islet Organoids Phantoms *in vitro*

**FIGURE 4:**
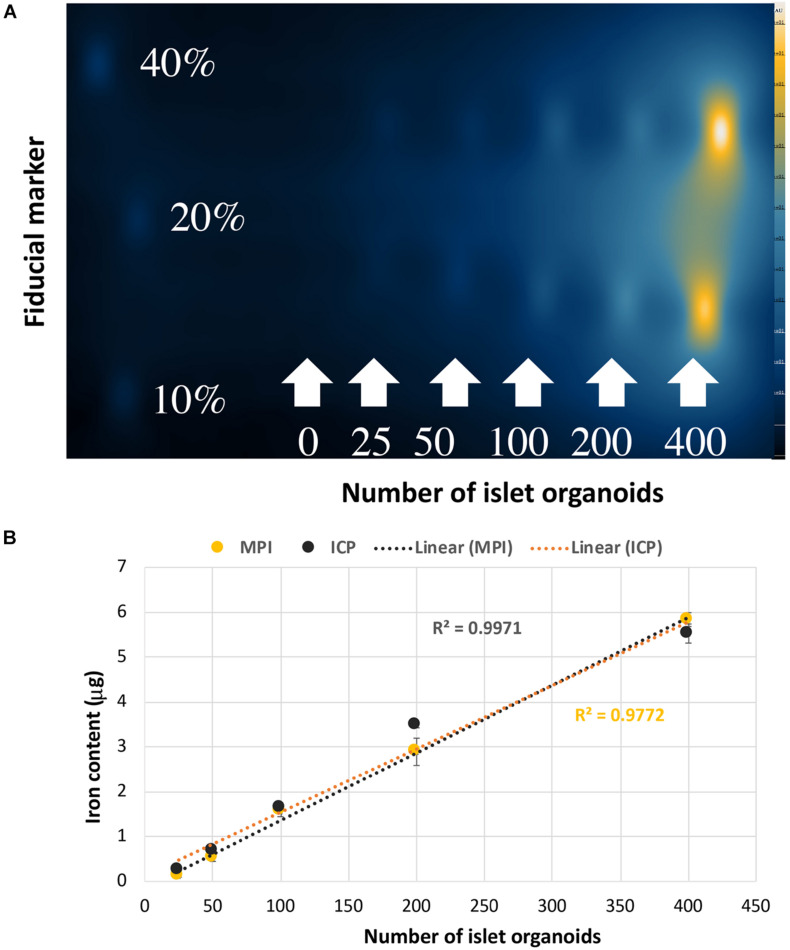
Islet organoids phantoms imaging and iron content quantification. **(A)** MPI of the different number of SPIO labeling islet organoids phantom. **(B)** Iron content of the islet organoids phantoms measured by MPI (*R*^2^ = 0.9971) and ICP-OES (*R*^2^ = 0.9772) both correlated with the number of labeled islet organoids. Data are represented as mean ± SD.

*In vitro* MPI showed the signal intensity of phantoms increased with increasing number of islet organoids (Figure 4A). The iron content was inferred from the MPI signal by quantitative analysis of the hand-drawn ROIs of MPI intensity using VivoQuant, calibrated against a 40% fiducial marker of known iron content (2.2 μg of iron). A linear correlation was revealed between the number of islet organoids and the MPI signal (*R*^2^ = 0.997, *p* < 0.0001) (Figure 4B). In equivalent control cell populations without VivoTrax labeling, no MPI signal was detected. Likewise, ICP-OES results showed that a linear correlation was found between the number of islet organoids and the iron content (*R*^2^ = 0.977, *p* < 0.0001) (Figure 4B), further confirmed the MPI quantification results.

### *In vivo* MPI *K-means*++ Algorithm Analysis and ICC Validation

**FIGURE 5:**
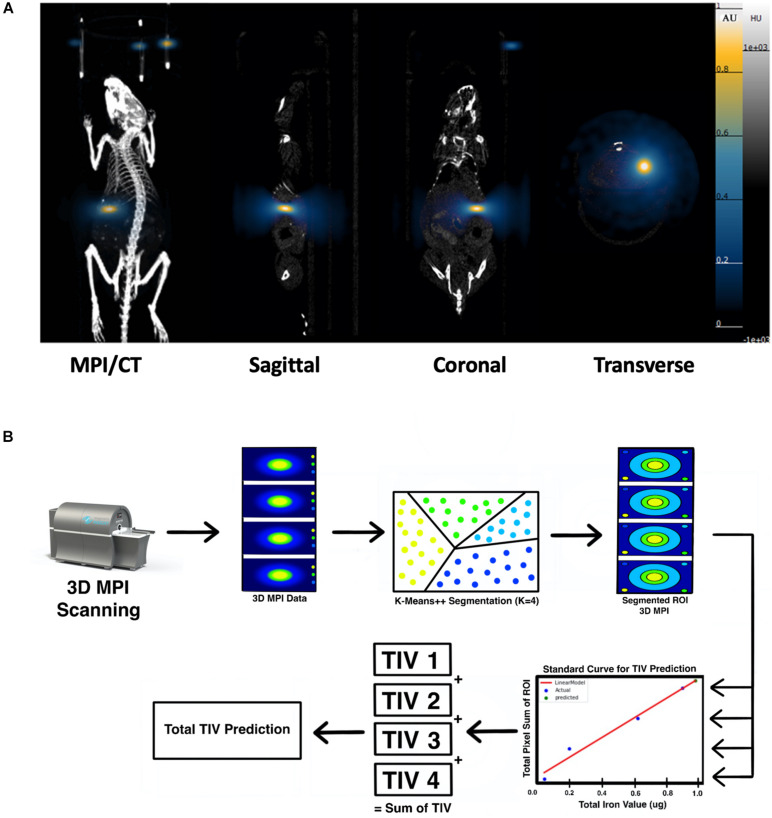
**(A)** 3D reconstructed MPI co-registered with microCT imaging of ViviTrax labeled islet organoids under the left kidney capsule of mouse on day 1 post-transplantation, all animals were placed in the prone position for both MPI and CT scanning. **(B)** Overview of the *K-means*++ algorithm for segmentation and TIV prediction.

Strong MPI signals representing labeled islet organoids were detected under the left kidney capsule from 3D images on the first day post-transplantation in all recipients (Figure 5A and Supplementary Video 2). *K-means*++ algorithm was applied for the 3D segmentations and TIV estimations on the ROIs from *in vivo* MPI (Figure 5B). Since MPI signal is not detectable in the absence of iron oxide nanoparticles, we did not observe any signal in control animals that did not receive the labeled graft Supplementary Video 3).

**FIGURE 6:**
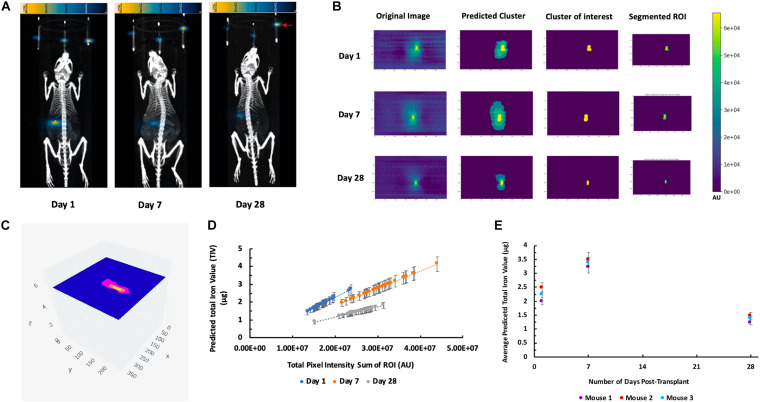
*K-means*++ segmentation and TIV prediction of longitudinal MPI of mice transplanted with islet organoids under the left kidney capsule. **(A)** Representative 3D MPI of mice days 1, 7, and 28 post Tx, all animals were placed in the prone position for both MPI and CT scanning. Red arrow shows the fiducial markers embedded in the animal scanning holder. **(B)** Single-slice output *K-means*++ segmentation of MPI ROI generated from original images through predicting clusters in MPI of recipient mice 1, 7, and 28 days post TX; Form left to right: original DICOM of 3D MPI; predicted clusters from *K-means*++ algorithm; mask of the predicted cluster of interest; real segmentation of original MPI using predicted mask. **(C)** Representative 3D volumetric rendering of *K-means*++ predictions of MPI ROIs on slices from a mouse post Tx. **(D)** TIV predictions from algorithm indicates a correlation between increasing total pixel sum per slice and the machine learning-generated TIV of days 1, 7, and 28 post Tx (*p* < 0.05). **(E)** Average of the TIV in the organoid transplants from days 1, 7, and 28 post transplantation in mice. Data are represented as mean ± SD (*p* < 0.05).

3D MPI were followed up at 7, and 28-day post-transplantation for these recipient mice (Figure 6A). Figure 6B depicts the output results from the single *k-means*++ segmentation of the ROI from longitudinal MPI scans of mice which received islet organoid transplants. Figure 6C and Supplementary Video 4 showed the 3D output of the signal *k-means*++ segmentation of the ROIs from an MPI scan of a recipient mouse. As is evidenced by the eventual loss of iron over the course of 28 days, which is visible both visually and through quantification *via* TIV estimation, the algorithm is able to trace the necessary ROI from the MPI image scans of mice and predict the TIVs which portray a linear trend in correlation with increasing total pixel sum of the ROI (Figure 6D, *p* < 0.05). An initial increase was observed in the MPI ROI size from day 1 to 7, with a decrease in overall ROI size by day 28 (Figures 6D,E, *p* < 0.05). Statistical evaluation *via* two-way repeated measures ANNOVA indicated significant difference between the predicted TIV from different days. These findings are reflected in the TIV prediction of the mice from different days in which there is an increase in TIV (and ROI size) from day 1 to 7 followed by a decrease from day 7 to 28. These results were fortified by ICC validation of the algorithm’s performance in comparison to a board-certified imaging specialist. A near-excellent and excellent ICC score of 0.898 and 0.927 for single and average measures, respectively, was determined through ICC validation (Table 1, *p* < 0.05). This indicated a high degree of agreement between TIV prediction of the algorithm and the imaging specialist.

**TABLE 1:** Intraclass correlation coefficient (ICC) validation of ROI segmentation and TIV prediction of the *K-means*++ based algorithm in comparison to an imaging specialist.

**Type**	**Interclass correlation**	**95% Confidence interval: lower bound**	**95% Confidence interval: upper bound**	***F*-test with true value 0: df**	***F*-test with true value 0: sig.**
Single measures	0.898	0.621	0.979	14	0.001
Average measures	0.927	0.546	0.973	14	0.001

*Top row: data for single measures.*

*Bottom row: data for average measures.*

### *Ex vivo* Immunohistochemistry

**FIGURE 7:**
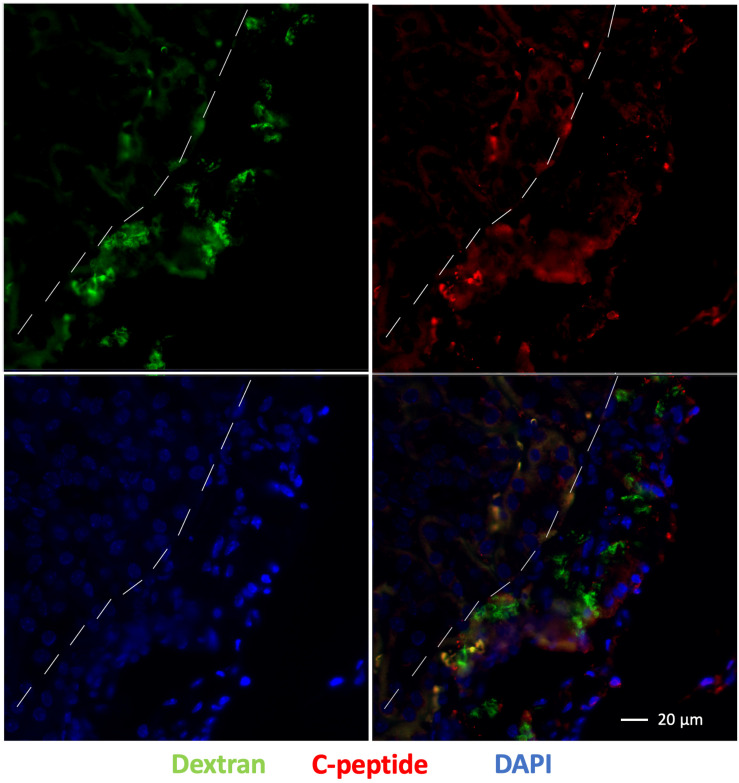
Fluorescence immunostaining of grafts under the kidney capsule 1 week after transplantation: dextran coating (green), C-peptide (red), and cell nuclei (DAPI, blue), magnification bar = 20 μm.

Immunofluorescence staining of the left kidney sections showed the presence of islet organoid grafts as confirmed by double staining for dextran and C-peptide 1-week post transplantation (Figure 7). Dextran expression was visible in these graft cells, which indicated that VivoTrax were retained in these transplanted cells (Figure 7). With the double immunostaining, these graft cells also expressed C-peptide, which demonstrated insulin biosynthesis in the graft organoids (Figure 7). This *ex vivo* data, consistent with *in vivo* MPI results, attest to the feasibility of labeling islet organoids using SPIONs and monitoring these functional islet organoids by MPI after transplantation.

## Discussion

Stem cell replacement therapy is a promising approach for the treatment of T1D. Studies demonstrated that hiPSC can become an unlimited, relatively safe (autologous-derived cells, thus devoid of rejection risk), and an efficient alternative source to generate insulin-producing islet organoids (Soejitno and Prayudi, 2011; Li et al., 2014; Maxwell et al., 2020). Methods to track islet organoids outcome after transplantation, however, are needed. MPI is an emerging tomographic technique that directly detects iron oxide nanoparticle tracers. These tracers are safe, non-toxic tracer, biocompatible and not normally found in the body, thus MPI images have exceptional contrast and high sensitivity. MPI imaging of these tracers provided an approach to track islet organoids and longitudinally monitor islet organoids *in vivo*. In this study, we successfully differentiated hiPSC-L1 into islet organoids and labeled islet organoids with SPIONs. Labeled islet organoids were highly effective at accumulation of SPIONs, immunostaining demonstrated nearly all of the cells within the islet organoids were labeled with SPIONs. MPI has a detection sensitivity of as few as 25 islet organoids *in vitro*. The quantification demonstrates a linear correlation between MPI signal intensity and the number of labeled islet organoids. We further found that the estimated iron content from the MPI image was consistent with the iron content measured by ICP. This demonstrates that MPI is able to quantify SPIONs-labeled islet organoids *in vitro*, which provides evidence that MPI may be able to track and quantify islet organoids *in vivo*. We used 800 labeled islet organoids per transplant to establish the feasibility of MPI detection *in vivo*. MPI 3D images with a co-registered CT show strong signals were detected under the left kidney capsule on the first day in all recipients that represented labeled islet organoids.

To monitor reproducibly the transplanted islet organoids longitudinally and analyze the ROIs from the MPI scans of mice, the canonical unsupervised ML algorithm of *k-means*++ was employed in conjunction with a linear regression based standard curve model. This algorithm is different from that which we applied previously to 2D MPI scans of transplanted islets, because it applies the algorithm to multiple layers of a 3D MPI scan (Hayat et al., 2020). This signal is indicative of the current nanoparticle content inside the mice which is evidence of the islet organoid cell content at the time of MPI scanning. Through segmentation and TIV prediction on the MPI data of the mice from days 1, 7, and 28, the algorithm was able to monitor the transplanted islet organoids longitudinally with indications on total iron content within subjects in a 3D manner.

The reasons why we chose VivoTrax for this study was because this tracer has been widely used for MPI. There are other SPIONs available, Feraheme (ferumoxytol) is another SPIONs product that is clinically used for iron anemia/deficiency and may have potential for MPI, although likely with less sensitivity due to its smaller size (Bulte, 2019). It was unexpected that there would be an increase in TIV (and ROI size) of the labeled transplants from day 1 to 7. The possible explanations for this finding include environmental induced degradation of the dextran coating of the VivoTrax, which may increase the total signal intensity, decreasing the peak signal intensity. Dynamic light scattering (DLS) studies have demonstrated that an increase in the nanoparticle size of degraded VivoTrax from 75 to 330 nm, which suggests the removal of the dextran coating resulted aggregation of the nanocrystal core *in vitro* (Guzy et al., 2020). Another factor that may contribute to this signal increase could be cell division triggered aggregation and releasing of the iron oxides nanostructure from encapsulated VivoTrax in the proliferating stem cell differentiated islet organoids. Possible explanation for the increase in TIV and ROI size might also be in relation with restructuration of the graft upon transplantation: the cell cluster break down and migrate to occupy the space under the kidney capsule, allowing vascularization and innervation of the grafts. Davalli et al. (1996) investigated syngeneic islets transplantation under the kidney capsule in a murine model. Grafts were harvested 1, 3, 7, and 14 days after transplantation and analyzed for morphology and insulin content. Their results showed that substantial damage in islet grafts was found on days 1 and 3 with apoptotic nuclei and necrotic cores; Tissue remodeling occurred with stable graft appearance on day 14; Graft insulin content decreased on day 1 and fell even further on days 3 and 7. We do agree that there are differences between *in vivo* imaging and histological evaluations, hence, we want to emphasize this is the first study on MPI of stem cell differentiated islet organoids transplantation, which is similar but still different from islet transplantation. In addition, measurement of labeled islet organoids in our study relies on imaging of the aforementioned nanoparticles, whereas Davalli et al. (1996) evaluated and quantified graft loss based on factors such as *ex vivo* morphology and insulin content/mRNA expression. This initial observation, irrespective of underlying mechanism, demonstrates the power of our data analysis algorithm for *in vivo* MPI.

Our method faces limitations as it cannot be applied to MPI scans that do not have the reference fiducial markers in the proper spatial orientation. Furthermore, it only takes into account three concentrations of fiducial markers in order to generate the standard curve with which the TIV is predicted. Future studies involving deep learning can potentially resolve these limitations by training on known TIV of diverse phantoms and their associated ICP values in order to predict the unknown TIV of a new ROI (Hayat and Wang, 2020). Nonetheless, the current algorithm provides a standardized, automated method for TIV analysis of transplanted organoids *in vivo*. This has implications for future studies in which the therapeutic effect of the transplanted islet organoids can be observed in diabetic mice, and such ML algorithms will be employed to accurately monitor the labeled iron signals from the organoid cell clusters longitudinally. This also has other implications in applying AI to monitor different cell-based therapies such as pancreatic islet transplantation, chimeric antigen receptor (CAR), T-Cell therapy, and other cellular approaches to treating diseases.

## Conclusion

In this study, we demonstrated the feasibility of longitudinal *in vivo* tracking and quantifying implanted islet organoids grafts for 28 days using MPI and ML algorithm. We believe that MPI could play an important role in monitoring the grafts, by directly imaging of the graft itself. In future studies, we plan to transplant islet organoids in a diabetic mouse model and verify and improve the *K-mean*++ algorithm for unbiased quantification of *in vivo* MPI.

## Data Availability Statement

The original contributions presented in the study are included in the article/Supplementary Material, further inquiries can be directed to the corresponding author/s.

## Ethics Statement

The animal study was reviewed and approved by Institutional Animal Care and Use Committee (IACUC) at the Michigan State University.

## Author Contributions

AS, HH, SL, JB, BD, MG, NT, and JD researched the data. HH and AS led the data analysis. ET, JB, BD, MG, and JG participated in data analysis. AS, HH, WL, AAl, and AAg participated in drafting the manuscript. PW conceived the idea, designed the study, drafted the manuscript and is the guarantor of this work and, as such, had full access to all the data in the study and take responsibility for the integrity of the data and the accuracy of the data analysis. All authors contributed to the article and approved the submitted version.

## Conflict of Interest

JG was employed by company Magnetic Insight Inc. The remaining authors declare that the research was conducted in the absence of any commercial or financial relationships that could be construed as a potential conflict of interest. The reviewer RS declared a shared affiliation with the authors to the handling editor at the time of review.

## Publisher’s Note

All claims expressed in this article are solely those of the authors and do not necessarily represent those of their affiliated organizations, or those of the publisher, the editors and the reviewers. Any product that may be evaluated in this article, or claim that may be made by its manufacturer, is not guaranteed or endorsed by the publisher.

## Footnotes

1
https://imagej.net/Fiji
