# Supplementary material for: 3D in vivo Magnetic Particle Imaging of Human Stem Cell-Derived Islet Organoid Transplantation Using a Machine Learning Algorithm
Source: Front Cell Dev Biol. 2021 Aug 12;9:704483. doi: 10.3389/fcell.2021.704483 (PMC8397508; doi:10.3389/fcell.2021.704483)
Supplement: Supplementary file 1 [file Data_Sheet_1.docx]

Supplementary Material

3D *in vivo* magnetic particle imaging of human stem cell-derived islet organoid transplantation using a machine learning algorithm

**Aixia Sun^1,2 †^, Hasaan Hayat^1,3 †^, Sihai Liu^1,2,4^, Eliah Tull^5^, Jack Owen Bishop^1,6^, Bennett Francis Dwan^1,7^, Mithil Gudi^1,3^, Nazanin Talebloo^1,8^, James Raynard Dizon^9^, Wen Li^10,11^, Jeffery Gaudet^11,12^, Adam Alessio^11,13^, Aitor Aguirre^11^, Ping Wang^1,2*^**

^1^ Precision Health Program, Michigan State University, East Lansing, MI, USA.

^2^ Department of Radiology, College of Human Medicine, Michigan State University, East Lansing, MI, USA.

^3^ Lyman Briggs College, Michigan State University, East Lansing, MI, USA.

^4^ Department of Orthopedics, Beijing Charity Hospital, Capital Medical University, Beijing, China.

^5^ City University of New York, Medgar Evers College, Brooklyn, NY, USA.

^6^ Department of Neuroscience, College of Natural Science, Michigan State University, East Lansing, MI, USA.

^7^ College of Natural Science, Michigan State University, East Lansing, MI, USA.

^8^ Department of Chemistry, College of Natural Science, Michigan State University, East Lansing, MI, USA.

^9^ Department of Radiology, UT Southwestern Medical Center, Dallas, TX, USA.

^10^ Department of Electrical and Computer Engineering, College of Engineering, Michigan State University, East Lansing, MI, USA.

^11^ Institute for Quantitative Health Science and Engineering (IQ), Department of Biomedical Engineering, Michigan State University, East Lansing, MI, USA.

^12^ Magnetic Insight Inc. Alameda, CA, USA.

^13^ Department of Computational Mathematics, Science, and Engineering (CMSE), College of Engineering, Michigan State University, East Lansing, MI, USA.

**^†^** These authors have contributed equally to this work and share first authorship

*** Correspondence:** Ping Wang

[wangpin4@msu.edu](mailto:wangpin4@msu.edu)

**Materials and Methods**

*Quantitative RT-PCR (qRT-PCR)*

After undergoing 3D differentiation, islet organoids were checked for hormone markers of human insulin and glucagon at mRNA and protein levels. Total RNA was extracted from embryoid bodies and islet organoids to analyze gene expression of insulin and glucagon using the RNeasy Mini Kit (Qiagen, Thermo Fisher Scientific, MA) with DNase treatment (Invitrogen, Thermo Fisher Scientific, MA), and cDNA was synthesized using miScript II RT kit (Qiagen). qRT-PCR reactions were performed with QuantiTect SYBR® Green PCR Kits (Qiagen) and analyzed using ∆∆Ct methodology. The forward and reverse primer of insulin used for quantitative PCR were 5’-GCTGGAGAACTACTGCAACTA-3’ and 5’-GCTGGTTCAAGGGCTTTATTC-3’. The forward and reverse primer of glucagon used for quantitative PCR were 5’-ATCTTCACAACATCACCTGCTA-3’ and 5’-GGCCTCAGAATACACCTCTTAAA-3’ respectively. The TATA binding protein (TBP) was used as a housekeeping control. Primers were purchased from Integrated DNA Technologies, Coralville, IA).

*Comparative analysis of total iron value (TIV) of labeled islet organoids between day 1 and day 7 in culture*

On day 1 post cell labeling, 200 of VivoTrax labeled islet organoids in 80 μl PBS were imaged using an MPI scanner (MOMENTUM MPI, Magnetic Insight Inc., Alameda, CA). Each 2D MPI image was acquired with parameters of a field-of-view (FOV) of 6 cm×12 cm, a 5.7 T/m selection field gradient, a drive field strength of 20 mT peak amplitude and a 45.0 kHz drive frequency. The scans were set up such that three 1μl fiducial markers, of increasing concentrations (0.55 μg/μl, 1.1 μg/μl, 2.2 μg/μl) were stacked vertically to the left of the main cell phantoms. This allowed us to generate a standard curve using the known total iron values of these reference markers. Upon segmenting the main ROIs and these fiducial markers using the *K-means++* algorithm, we generated a standard curve using the extracted “Total Pixel Sum” of each fiducial marker and their corresponding TIV. Then, the total pixel sum of the main phantom ROI was used to calculate its unknown TIV using this generated standard curve.

Supplemental Figure 1


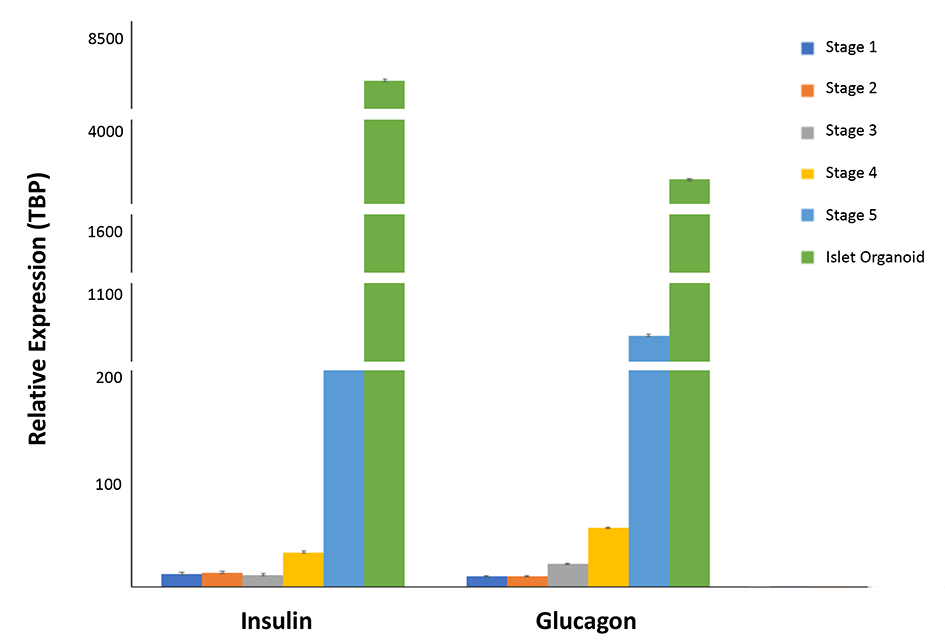


Supplemental Fig. 1: Quantitative RT-PCR analysis of gene transcripts in the embryoid body at 5 stages under differentiation and in islet organoids. Results shown relative to the house keeping control TBP gene expression. Data are represented as mean ± SD.

Supplemental Figure 2


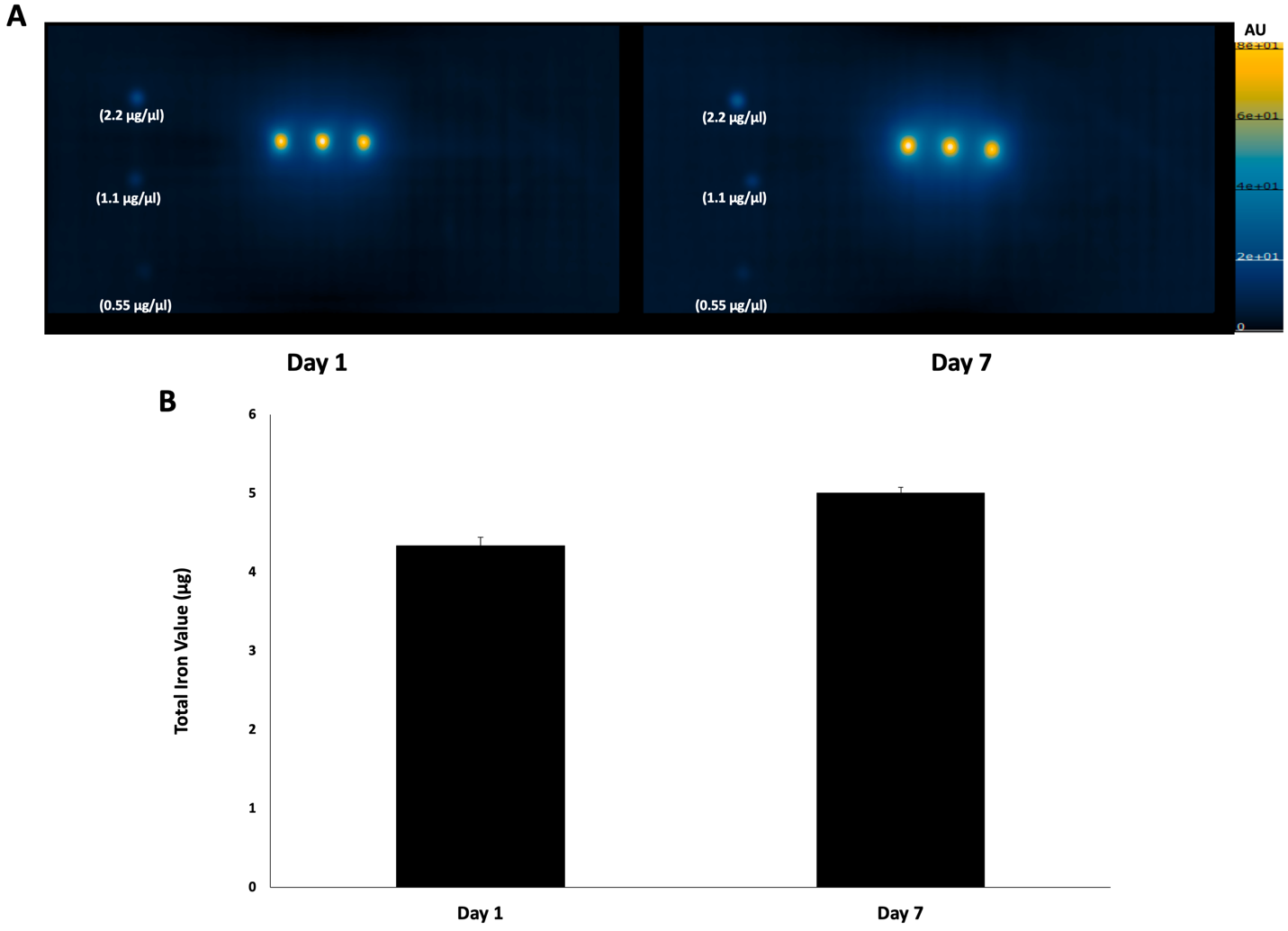


Supplemental Fig. 2: A. MPI signal intensities of *in vitro* cultured VivoTrax labeled islet organoids on day 1 and day 7. B. Total iron values calculated based on the MPI intensities for in vitro cultured islet organoids (n = 3).

Supplemental Video 1

Representative projection imaging of confocal microscopy of islet organoids stained for immunofluorescence to determine expression of insulin and glucagon.

https://drive.google.com/file/d/1DOiVGFEeGV4COuZJTS3A9eGnEcSwJ_9X/view?usp=sharing

Supplemental Video 2

Representative 3D MPI of experimental group of mice day 1, 7, and 28 post Tx.

https://drive.google.com/file/d/1_ajq4N0sid-SWi7VQZ608oqnK6DhvL2v/view?usp=sharing

Supplemental Video 3

Representative 3D MPI of control group of mice day 1, 7, and 28 post Tx.

https://drive.google.com/file/d/1iNra_08NfXuObtsGW-fwSR4X0xd0UAUX/view?usp=sharing

Supplemental Video 4

3D slices output *K-means++* segmentation of MPI ROIs from mouse post Tx.

https://drive.google.com/file/d/1NNImzMkfmz1EWk3jZNZuE-fttiFq1xrp/view?usp=sharing
